# Identification of a prognosis-related ceRNA network in cholangiocarcinoma and potentially therapeutic molecules using a bioinformatic approach and molecular docking

**DOI:** 10.1038/s41598-022-20362-w

**Published:** 2022-09-28

**Authors:** Xiaoling Gao, Wenhao Zhang, Yanjuan Jia, Hui Xu, Yuchen Zhu, Xiong Pei

**Affiliations:** 1 The Medical Laboratory Center, Hainan General Hospital, Haikou, 570311 China; 2 The First School of Clinical Medicine, Lanzhou University, Lanzhou, 730000 China; 3 NHC Key Laboratory of Diagnosis and Therapy of Gastrointestinal Tumor, Gansu Provincial Hospital, Lanzhou, 730000 China; 4 The Institute of Clinical Research and Translational Medicine, Gansu Provincial Hospital, Lanzhou, 730000 China; 5 Gansu University of Chinese Medicine, Lanzhou, 730000 China; 6 The Second Clinical Medical College, Lanzhou University, Lanzhou, 730000 China

**Keywords:** Cancer, Oncology

## Abstract

Cholangiocarcinoma (CCA) is a highly malignant disease with a poor prognosis, and mechanisms of initiation and development are not well characterized. It is long noncoding RNAs (lncRNAs) acting as miRNA decoys to regulate cancer-related RNAs in competing endogenous RNA (ceRNA) networks that suggest a possible molecular mechanism in CCA. The current study aims to find potential prognosis biomarkers and small molecule therapeutic targets based on the construction of a CCA prognosis-related ceRNA network. A transcriptome dataset for CCA was downloaded from the TCGA database. Differentially expressed lncRNAs (DElncRNAs), DEmiRNAs and DEmRNAs were identified based on the differential expression and a DEceRNA network was constructed using predicted miRNA-lncRNA and miRNA-mRNA interactions. Heat maps, PCA analysis, and Pathway enrichment analysis and GO enrichment analysis were conducted. The prognostic risk model and molecular docking were constructed based on identified key ceRNA networks. A DElncRNA-miRNA-mRNAs network consisting of 434 lncRNA-miRNA pairs and 284 miRNA-mRNA pairs with 200 lncRNAs, 21 miRNAs, and 245 mRNAs was constructed. There were three lncRNAs (AC090772.1, LINC00519, and THAP7-AS1) and their downstream mRNAs (MECOM, MBNL3, RCN2) screened out as prognostic factors in CAA. Three key networks (LINC00519/ hsa-mir-22/ MECOM, THAP7-AS1/hsa-mir-155/MBNL3, and THAP7-AS1/hsa-mir-155/RCN2) were identified based on binding sites prediction and survival analysis. A prognostic risk model was established with a good predictive ability (AUC = 0.66–0.83). Four anticancer small molecules, MECOM and 17-alpha-estradiol (−7.1 kcal/mol), RCN2 and emodin (−8.3 kcal/mol), RCN2 and alpha-tocopherol (−5.6 kcal/mol), and MBNL3 and 17-beta-estradiol (−7.1 kcal/mol) were identified. Based on the DEceRNA network and Kaplan–Meier survival analysis, we identified three important ceRNA networks associated with the poor prognosis of CCA. Four anti-cancer small molecules were screened out by computer-assisted drug screening as potential small molecules for the treatment of CCA. This study provides theoretical support for the development of ceRNA network-based drugs to improve the prognosis of CCA.

## Introduction

Cholangiocarcinoma (CCA) is a rapidly progressing cancer with an extremely poor prognosis. CCA patients are usually diagnosed in the later stage due to the lack of recognizable symptoms and sensitive early diagnosis measures. CCA is categorized as intrahepatic, perihilar, and distal CCA^1^. According to statistics from WHO, the incidence of CCA has increased worldwide over the past decades, especially in eastern Asia including China, South Korea, and Thailand^2^. The incidence of CCA in China has exceeded 6 deaths per 100,000 people for many years^3^. The current primary treatment for CCA is resection with regional lymphadenectomy and EBRT with concurrent fluoropyrimidine regional therapy^4^. Targeted therapy has recently generated modest benefits; however, CCA is highly heterogeneous with redundancy of mutation such that only a small proportion of patients bearing drug-targeted genome alterations benefit from molecular-targeted therapies. Therefore, there is a great urgency to elucidate the molecular mechanisms of CCA, identify new diagnostic biomarkers and therapeutic targets, as well as develop effective therapeutic medicine for CCA.

Although pseudogenes have long been thought to be nonfunctional, recent studies proved that some expressed pseudogenes equipped with transcripts biologically function^5^. Poliseno et al. reported that the pseudogene PTENP1 regulated the level of PTEN in human cancer cells and exerted a growth inhibitory effect^6^. Some expressed pseudogene transcripts have biological functions, such as a subclass like functional long noncoding RNAs (lncRNAs). lncRNAs regulate gene expression in multiple ways: lncRNAs interact with DNA, RNA, or proteins, such as RNA–DNA–DNA triplexes, to affect the structure and function of chromatin so that regulate nearby and distant genes^7^. Besides, lncRNAs could act as competitive endogenous RNAs (ceRNA) to bind microRNAs (miRNAs), thus attenuating their regulatory role in promoting or hampering the translation of downstream protein-coding messenger RNAs (mRNAs), which may indeed have important functional roles in various human cancers^8^.

ceRNAs have been used to clarify the occurrence and development of CCA, and some lncRNAs and miRNAs have been demonstrated to serve as potential diagnostic markers and therapeutic targets in CAA. Some research worked on the ceRNA networks in CAA development, but with discrepant conclusions. The research from Dongkai Zhou et al. and Zhichen Kang et al. focused on intrahepatic CCA and found lncRNA HULC or MME-AS1 with other 6 hub mRNAs, respectively, which was related to the prognosis of ICC^9,10^. Junyu Long et al. identified 26 lncRNAs, 3 miRNAs, and 13 mRNAs as prognostic biomarkers for patients with CCA^11^. Wang Xiang et al. found that three lncRNAs COL18A1-AS1, SLC6A1-AS1, and HULC are associated with overall survival^12^. Based on all these discrepancies, we intended to identify a comprehensive ceRNA network based on the updated database. And, with the aim to verify the potential biomarkers and the possible binding way of proteins and anti-cancer small molecules, molecular docking validation was performed.

In the present study, in order to identify the prognosis-related ceRNA networks and the potential anti-cancer drug treatments for CCA, we screened differentially expressed RNAs to establish DElncRNA-miRNA-mRNA networks and performed survival analysis and binding-sites prediction. We constructed three key prognosis-related ceRNA networks. And we used molecular docking to virtual screen out four anti-cancer small molecules. Moreover, our study contributes to understanding the regulatory mechanism of CCA better and gives valuable insight into the development of potential target drugs for CCA.

## Materials and methods

### Data source and processing

**Figure 1:**
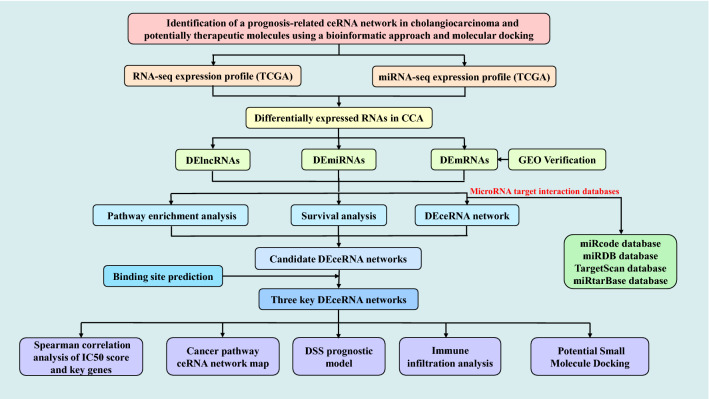
Flow chart of the methodology of the study.

Figure 1 shows the workflow schematic of this study. The cancer genome atlas (TCGA), a landmark cancer genomics program, molecularly characterized over 20,000 primary cancer and matched normal samples spanning 33 cancer types. We identified and downloaded RNA-Seq profiles (lncRNAs, miRNAs, and mRNAs) and clinical data for CCA. There were 8 normal mRNA samples and 33 tumor samples in the 41 cases of CCA, and 8 normal samples and 33 tumor samples in the 41 miRNA samples.

### Differential expression analysis

Based on the standard of |logFC|≥ 2, adjusted p-value < 0.05, the differentially expressed mRNAs, miRNAs, and lncRNAs were analyzed using the “EdgeR” package in R software^13^. The miRNA-lncRNA interactions were predicted and confirmed in the miRcode database^14^ and miRNA-mRNA interactions were predicted and confirmed with the miRDB database, TargetScan Human database**,** and miRtarBase database^15–17^. Differential expression analysis of 8 paired samples was performed with the Wilcoxon test.

### Construction of a ceRNA network and Kaplan–Meier survival analysis

According to the endogenous competitive network hypothesis, relationship pairs with the same expression levels of DElncRNAs and DEmiRNAs are ignored. The rest of the differential lncRNA-miRNA and miRNA-mRNA pairs were selected and a ceRNA network was constructed and visualized with Cytoscape 3.7. Survival data available from the TCGA database and survival analysis was further analyzed by the “survival” package, which generated survival curves for DElncRNAs (p-value < 0.05). In addition, all DEmRNAs were entered into the StarBase database and the ones associated with significant survival (p-value < 0.05) were selected^18^, and a survival-related ceRNA network was generated. The binding sites of lncRNAs and mRNAs with their corresponding miRNAs were predicted based on their sequences with miRanda^19^ and the TargetScan database.

### PCA analysis and functional enrichment analysis

The heatmaps and PCA analysis were generated and visualized on Hiplot (https://hiplot-academic.com/)^20^. Pathway enrichment analysis was conducted based on the Reactome database using Web-based gene set analysis toolkit (WebGestalt, http://www.webgestalt.org/)^21,22^ and Gene Ontology (GO) analysis was conducted and visualized utilizing the R “clusterProfiler” package on Hiplot^23^. A p-value < 0.05 was regarded as statistically significant.

### Construction of a prognostic risk model

The raw counts and corresponding clinical information from the RNA sequencing data were downloaded from TCGA. The expression of these genes in CCA patients was extracted and the transcriptional information from the patients was matched with the accompanying clinical data. The least absolute shrinkage and COX regression algorithm were conducted by R “glmnet” and “survival” packages for candidate gene selection, using tenfold cross-validation, and the penalty parameter (λ) was determined using the minimum criteria. Finally, the candidate genes and their coefficients were used to construct the risk model. The formula to calculate the risk score was as follows: Risk score = ${\sum }_{i}^{n}Xi\times Yi$ (X: coefficients, Y: gene expression level). Patients were classified into high-risk and low-risk groups according to the median value of the risk score. Kaplan–Meier (KM) survival analysis with a log-rank test was utilized to make comparisons of the survival difference between the high and low-risk groups. Finally, the timeROC analysis was applied to perform the predictive accuracy of the risk model.

### Molecular docking and virtual screening

To verify the potential drug target of MBNL3, MECOM, RCN2, and potential small molecules, we obtained their related small molecules from the HERB database (http://herb.ac.cn/)^24^, obtained the protein structure from the AlphaFold database (https://alphafold.com/)^25^, predicted pockets of activity for drug binding of candidate proteins, and calculated the lowest free energy of binding between the candidate protein and small molecule through the AutoDock Vina soft. Finally, visualization was performed with Pymol^26,27^.

### Immune infiltration and spearman correlation analysis of IC50 scores and MECOM, MBNL3, and RCN2 expression

Immune infiltration analysis of the six survival-related genes was conducted on the Timer 2.0 database (http://timer.comp-genomics.org/)^28^. The results based on TIMER and CIBERSORT algorithms were retained. Spearman correlation analysis of the IC50 score was conducted using R “pRRophetic” package based on Genomics of Drug Sensitivity in Cancer (GDSC) database (https://www.cancerrxgene.org/)^29^.

### Ethical approval

Our study was based on open-access databases, TCGA and GEO. Users could download relevant data for free for research and publication purposes. The data from the patients who had consented to participate and ethical approval had been obtained for each study.

## Results

### Identification of differentially expressed RNAs

**Figure 2:**
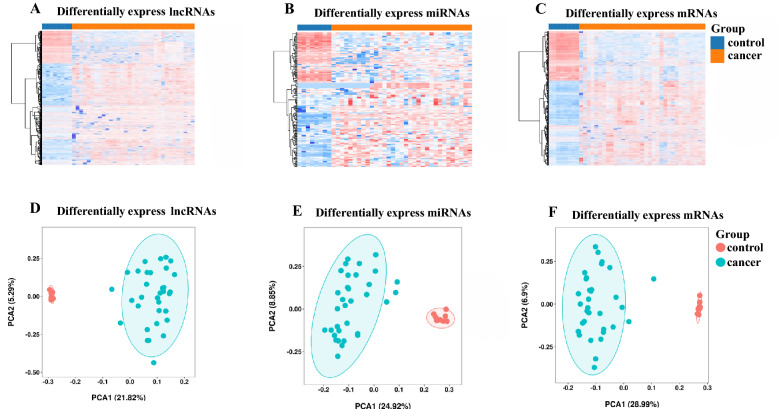
Data from the analysis of differential expression between CCA samples and control samples derived from the TCGA transcriptome. (**A**–**C**): Heat maps of 2771 DElncRNAs, 173 DEmiRNAs, and 6837 DEmRNAs from 33 CCA samples and 8 normal samples; the upper part of the heat map represents the sample, orange is the control group and blue is the cancer group. (**D**–**E**): Principal component analysis (PCA) of DElncRNAs, DEmiRNAs, and DEmRNAs. Red is the control group, green is the cancer group, and the circle is the 95% confidence interval. R “ggplot2” package version 3.3–6, < URL: https://ggplot2.tidyverse.org > .

**Figure 3:**
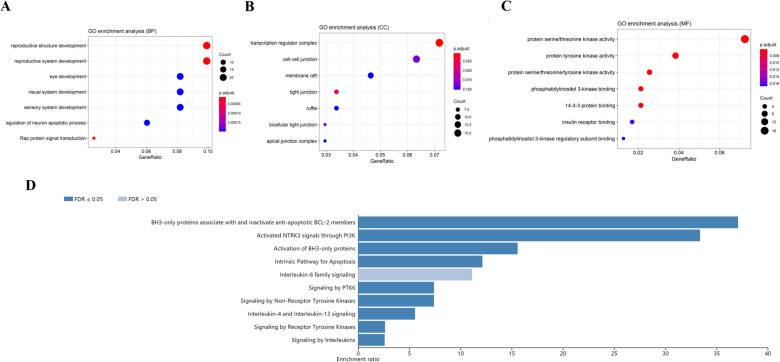
GO and Pathway enrichment analysis functional enrichment analysis of 6837 DEmRNAs differentially expressed between CCA samples and control samples derived from the TCGA transcriptome. (**A**–**C**): Cellular component, molecular function, and biological process enrichments for DEmRNAs in the network; the size of the dots represents the number of enriched genes, and the color represents p adjustment. (**D**): Pathway enrichment analysis of DEmRNAs in the network; the length represents the enrichment ratio, and the color depth represents the extent of FDR significance.

To illustrate the differentially expressed RNAs in CCA, we collected data from 33 CCA and 8 adjuvant normal tissues in the TCGA database and performed a differential expression (DE) analysis. We identified 6837 DEmRNAs (61.9% up-regulated and 38.1% down-regulated), 2771 DElncRNAs (69.3% up-regulated and 30.7% down-regulated) and 173 DEmiRNAs (63.6% up-regulated and 36.4% down-regulated). The heat maps showed that DElncRNAs (Fig. 2A), DEmiRNAs (Fig. 2B), and DEmRNAs (Fig. 2C) were differentially expressed between normal and cancer samples with obvious boundaries. The principal component analysis (PCA) analysis based on 3 types of DERNAs indicated that cancer samples and normal samples can be well isolated by PCA1 components, with an explanation rate of 21.82% (Fig. 2D), 24.92% (Fig. 2E), and 28.99% (Fig. 2F), which indicated that they were clearly distinguished from each other. Among the 33 CCA samples and 8 normal samples, there were 8 paired samples. To determine if there was a bias in the differential expression of RNAs between paired and unpaired samples, we conducted a differential analysis based on the paired samples. The results were all significant except for hsa-mir-155-5p (p-value = 0.13) (Fig. S1). Further, Pathway enrichment analysis and GO analysis were carried out to identify the enriched functions and signal pathways involving DEmRNAs. They were enriched in GO terms (Fig. 3A–C) like “reproductive structure development”, “transcriptional regulatory complexes”, “tyrosine, serine, threonine kinase activities” and “phosphatidylinositol 3-kinase activities”. While main pathways (Fig. 3D) were “activated NTRK3 signals through PI3K”, “activation of BH3-only proteins”, “intrinsic pathway for apoptosis”, “signaling by PTK6”, “signaling by Non-Receptor Tyrosine Kinases”, “IL-4 and IL-13 signaling” and “signaling by receptor tyrosine kinases”. These enriched pathways and function analysis results revealed that the development of CCA was closely related to tyrosine kinase activity, as well as immune responses.

### Differential ceRNA networks

**Figure 4:**
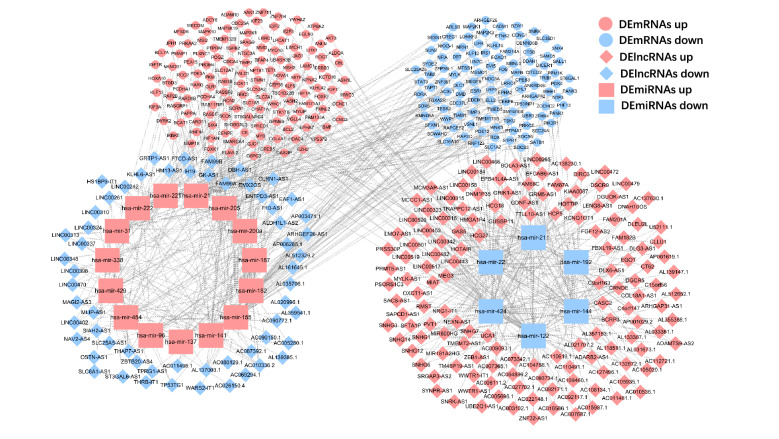
Construction of the ceRNA network on 200 lncRNAs nodes, 21 miRNAs nodes, and 245 mRNAs nodes with 434 lncRNA-miRNA pairs and 284 miRNA-mRNA pairs; the shape represents the type of RNA, blue means down-regulation in cancer samples, and red means up-regulation in cancer samples.

To better demonstrate the interactions and regulatory mechanisms between DElncRNAs, DEmiRNAs, and DEmRNAs, we constructed a complicated ceRNA regulatory networks diagram (Fig. 4) in which the lncRNA-miRNA pairs with the same ascending and descending relationship were deleted, including 200 lncRNAs nodes, 21 miRNAs nodes and 245 mRNAs nodes with 434 lncRNA-miRNA pairs and 284 miRNA-mRNA pairs. The prediction results of lncRNA-miRNA pairs (Supplementary Table S1) and miRNA-mRNA pairs (Supplementary Table S2) in the database are shown in the supplementary material.

### Kaplan–Meier survival analysis of DElncRNAs and DEmRNAs.

**Figure 5:**
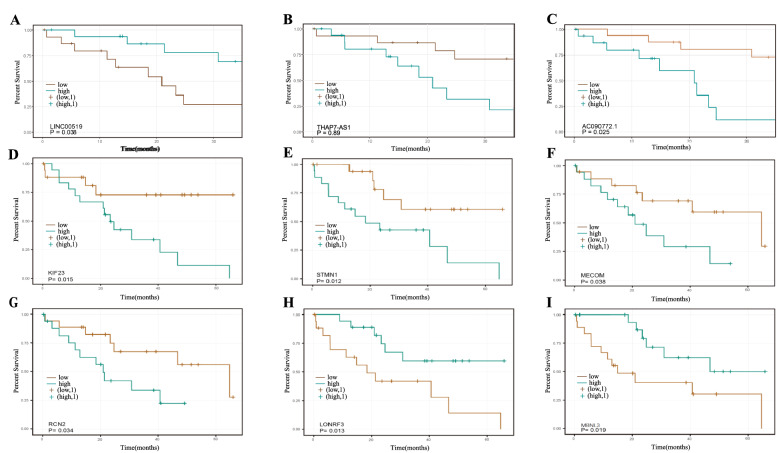
Kaplan–Meier survival analysis of survival-significant DElncRNAs using R “survival” package version 3.2–11, < URL: https://CRAN.R-project.org/package=survival > : LINC00519, THAP7-AS1, AC090772.1(**A**–**C**); survival-significant DEmRNAs: KIF23, STMN1, MECOM, RCN2, MBNL3, LONRF3(**D**–**J**).

Kaplan–Meier survival analysis was performed on all DElncRNAs and DEmRNAs in the DEceRNA network identified in our study to find the survival-related RNAs. The survival data from TCGA was used for Kaplan–Meier survival analysis. The median of gene expression was taken as a cutoff to partition the training set samples into high expression and low expression groups respectively, which were used to perform survival analysis. After the comprehensive survival analysis for 200 DElncRNA, LINC00519 (Long Intergenic Non-Protein Coding RNA 00,519), THAP7-AS1(THAP7 antisense RNA 1), and AC090772.1 (also named as ENSG00000264924) were identified as being related to the survival of CCA patients, as shown in Fig. 5. The increased expression level of LINC00519 indicated a worsening prognosis in CCA patients, (log-rank p-value = 0.038 Fig. 5A). In contrast, decreased THAP7-AS1 and AC090772.1 expression implied a worse survival time in CCA patients (log-rank p-value = 0.041 Fig. 5B, log-rank p-value = 0.025 Fig. 5C). Furthermore, three lncRNAs and their downstream DEmiRNAs and DEmRNAs-related ceRNA networks were selected for demonstration in supplemental materials. (Fig. S2).

Through survival analysis of 245 DEmRNAs, the expression of 6 mRNAs was significantly related to the survival rate. Among them, KIF23 (kinesin family member 23) (log-rank p-value = 0.015 HR = 3,69 Fig. 5D), STMN1 (stathmin 1) (log-rank p-value = 0.012 HR = 3,49 Fig. 5E), MECOM (MDS1 and EVI1 complex locus) (log-rank p-value = 0.038 HR = 2.80 Fig. 5F), RCN2 (Reticulocalbin-2) (log-rank p-value = 0.034 HR = 2.86 Fig. 5G) were highly expressed in cancer samples, while 2 were decreased in cancer samples: LONRF3 (LON peptidase N-terminal domain and ring finger 3) (log-rank p-value = 0.013 HR = 0.31 Fig. 5H), MBNL3 (muscle blind like splicing regulator 3) (log-rank p-value = 0.015 HR = 0.32 Fig. 5I). Especially, MECOM, MBNL3, and RCN2 appeared as downstream targets of the LINC00519, THAP7-AS1, and AC090772.1.

### Construction of key ceRNA networks and a cancer pathway ceRNA network map

**Figure 6:**
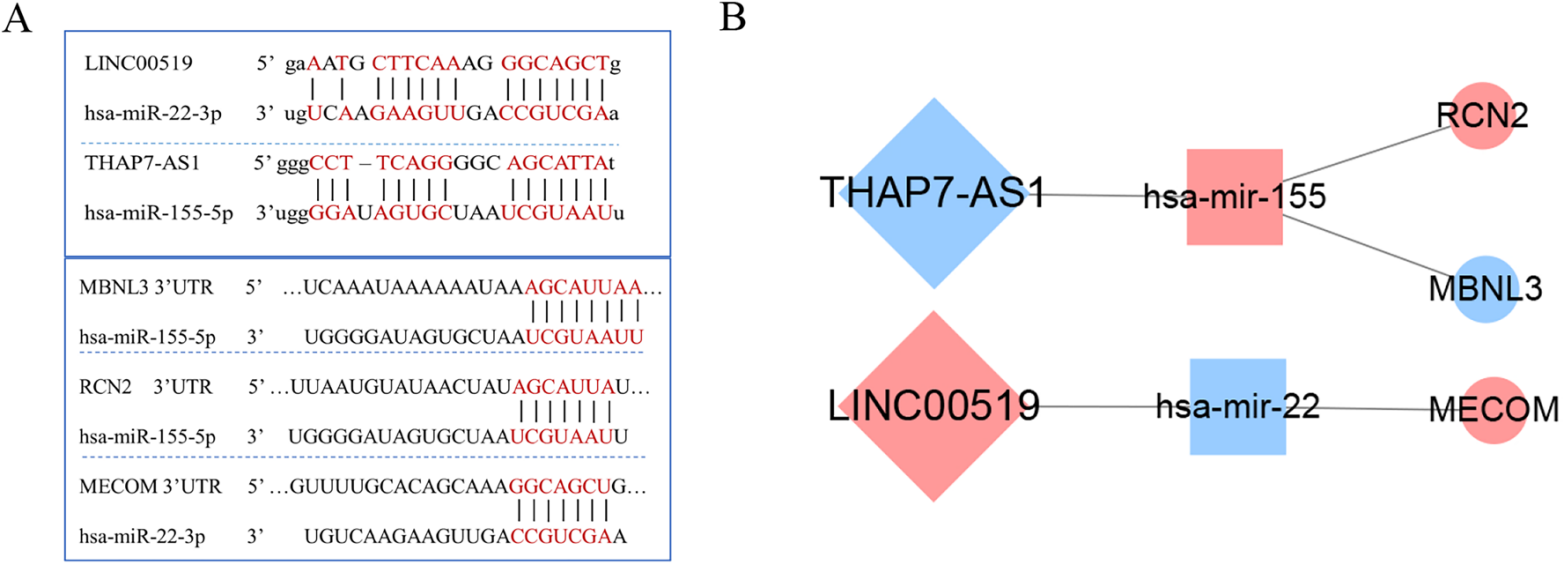
(**A**) Prediction of binding sites of lncRNA-miRNA and miRNA-mRNA in three key networks. (**B**) The construction of three ceRNA networks: LINC00519/ hsa-mir-22/MECOM, THAP7-AS1/hsa-mir-155/MBNL3, and THAP7-AS1/hsa-mir-155/RCN2; the shape represents the type of RNA, blue means down-regulation in cancer samples and red means up-regulation in cancer samples.

As shown in Fig. 6, we further constructed the key ceRNA networks related to survival rate. Four networks enrolled 3 lncRNA-miRNA pairs and 4 miRNA-mRNA pairs were obtained: LINC00519/ hsa-mir-22-3p/MECOM, THAP7-AS1/hsa-mir-429/MBNL3, THAP7-AS1/hsa-mir-155-5p/MBNL3 and THAP7-AS1/hsa-mir-155-5p/RCN2. Moreover, to demonstrate the direct binding reliability between lncRNA with miRNA as well as miRNA with mRNA, we checked the binding sites of lncRNAs and mRNAs with their corresponding miRNAs with miRanda and TargetScan. The lncRNAs and mRNAs had binding sites with the same miRNAs, suggesting that lncRNAs were likely to act as endogenous competitive RNAs to influence the expression of mRNAs (Fig. 6A). According to this, we selected three key pathways, including 2 lncRNAs, 2 miRNAs, and 3 mRNAs. Two lncRNA-miRNA pairs and 3 miRNA-mRNA pairs were obtained: LINC00519/ hsa-mir-22-3p/MECOM, THAP7-AS1/hsa-mir-155-5p/MBNL3 and THAP7-AS1/hsa-mir-155-5p/RCN2 (Fig. 6B). In CCA patients, the strongly reduced expression of THAP7-AS1 decreased the expression of MBNL3 and increased RCN2 expression. Similarly, the increased expression of LINC00519 hindered the expression of the downstream miRNA, which led to the overexpression of MECOM and appeared to affect the behavior of tumor cells. A mechanism map was drawn with MECOM, MBNL3, and RCN2, three key genes in the three key ceRNA networks we analyzed, by consulting a large number of documents (Fig. S3).

### Construction of disease-specific survival (DSS) prognostic risk model based on lncRNAs and genes in key ceRNA networks

**Figure 7:**
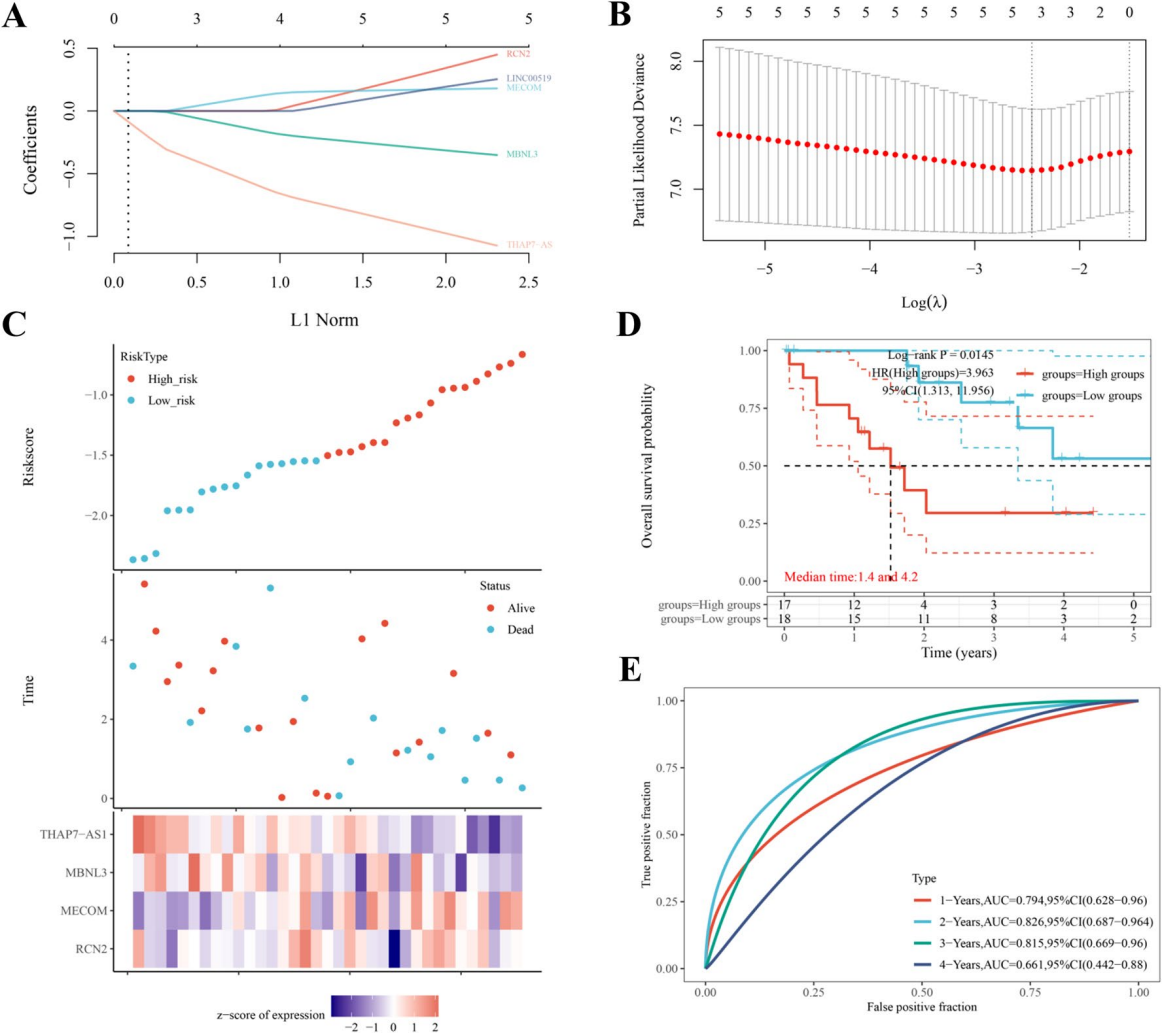
Construction of a prognostic risk model of five key RNAs by lasso regression and multifactorial cox using R “glment” package version 4.2–4, < URL: https://glmnet.stanford.edu > . (**A**): scatter plot of the five RNA expression levels from low to high, with different colors representing different expression groups; (**B**) The relationship between partial likelihood deviation and log (l) was plotted using the Lasso Cox regression model. (**C**): Scatter plot distribution of survival time and survival status corresponding to sample RNA expression and expression heat map. (**D**): Overall survival probabilities of the four RNAs in different expression groups; the Log-rank p-value, HR, and 95% confidence interval are marked above it; (**E**): the below are ROC curves predicting 1-, 2-, 3- and 4-year survival based on the risk score.

To explore the prognostic value of LINC00519, THAP7-AS1, MECOM, MBNL3, RCN2 in these ceRNA key networks, a DSS prognostic risk model was established as shown in Fig. 7, and the risk score = (0.593) * RCN2 + (0.1334) * LINC00519 + (0.1604) * MECOM + (-1.44) * THAP7-AS1 + (-0.2911) * MBNL3. Patients were separated into high-risk and low-risk groups according to the median risk score point. Firstly, the expression levels were demonstrated after differential expression analysis. The results showed higher expression of LINC00519, MECOM, RCN2, and lower expression of THAP7-AS1, and MBNL3 in tumors compared to the adjuvant tissues (Fig. 7A). The expression trends were consistent with the differential ceRNA network regulation strategy (Fig. 3). Secondly, four key RNAs were obtained through Cox regression analysis for the establishment of the final prognostic model (Fig. 7B). The relationship between the expression of these four key RNAs and survival is shown in Fig. 7C. In the overall survival probability, the high-risk and low-risk groups showed a significant difference in survival with a p-value of 0.0145, HR of 3.963, and a 95% CI (1.313, 11.956), and the median time between high-risk and low-risk groups was 1.4 years and 4.2 years (Fig. 7D). The AUC (1 ~ 4 years) was 0.66 ~ 0.83 (Fig. 7E) which indicated good predictive performance.

### Molecular docking and virtual screening

To find potentially approved drugs that target MECOM, RCN2, and MBNL3, we screened the herb database for related small molecules with anti-cancer effects.

**Figure 8:**
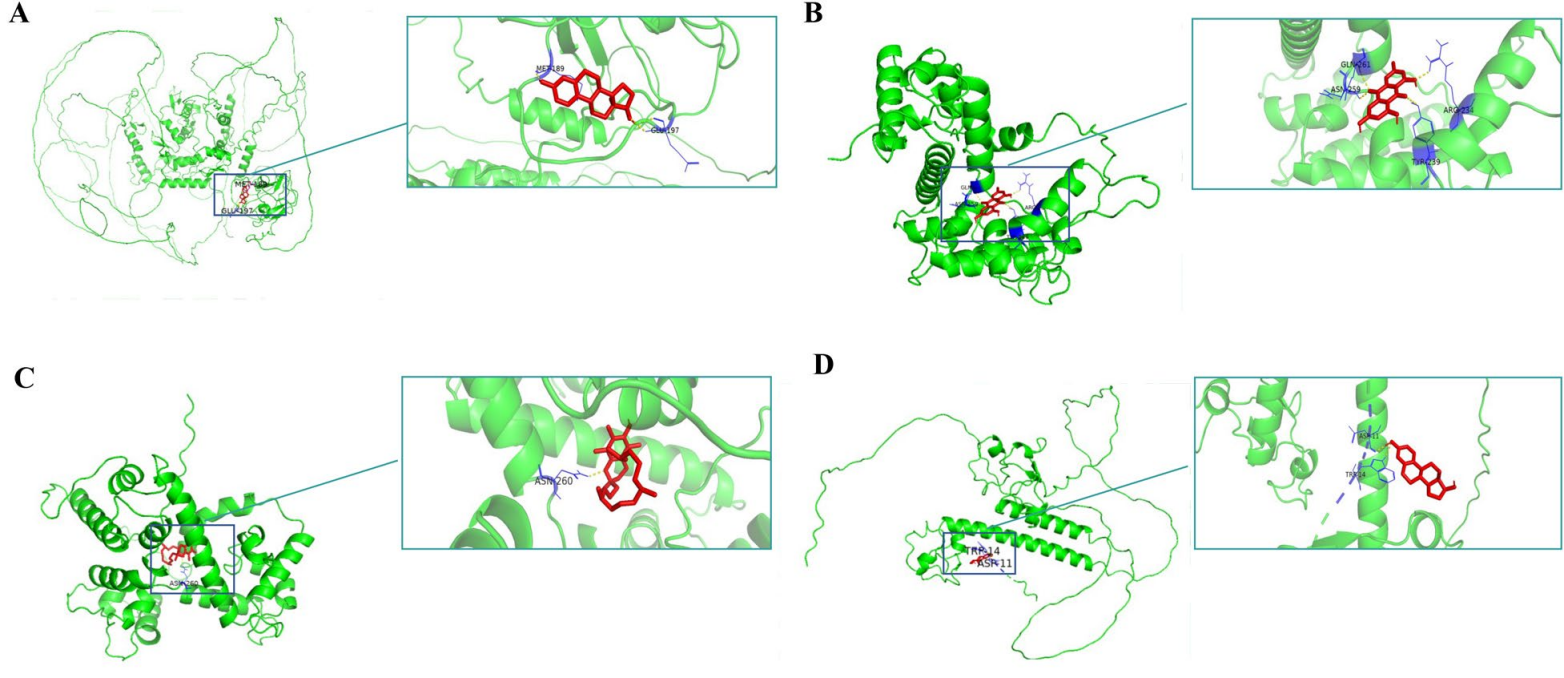
Molecular docking results for MECOM, RCN2, MBNL3. (**A**): A 3D view of the best-selected conformation of MECOM and 17-alpha-estradiol. (**B**): A 3D view of the best-selected conformation of RCN2 and emodin. (**C**): A 3D view of the best-selected conformation of RCN2 and alpha-tocopherol. (**D**): A 3D view of the best-selected conformation of MBNL3 and 17-beta-estradiol. Green color: protein; red color: small molecules; yellow color: conventional hydrogen bonds.

We visualized binders with lowest binding affinity (kcal/mol) after molecular docking: MECOM and 17-alpha-estradiol (−7.1 kcal/mol), RCN2 and emodin (−8.3 kcal/mol), RCN2 and alpha-tocopherol (-5.6 kcal/mol), MBNL3 and 17-beta-estradiol (−7.1 kcal/mol). The binding sites and interactions are displayed in Fig. 8. Based on the 3D view of the best-selected conformations, three, four, one, and two hydrogen bonds were found in the binding of MECOM and 17-alpha-estradiol (Figs. 8A), RCN2 and emodin (Figs. 8B), RCN2 and alpha-tocopherol (Figs. 8C), MBNL3 and 17-beta-estradiol (Figs. 8D).

### Spearman correlation analysis of the IC50 score and MECOM, MBNL3, and RCN2 expression

The expression level of MECOM was negatively correlated with the AZ628 IC50, as was the relationship between RCN2 and paclitaxel, and MBNL3 and crizotinib (Fig. S4). As the expression levels of MECOM, RCN2 and MBNL3 increased, the IC50 of AZ68, paclitaxel and crizotinib in CCA decreased. This indicated increasing sensitivity to AZ68 and paclitaxel in CCAs with high MECOM and RCN2 expression, while in CCAs with low MBNL3 expression, patients were resistant to crizotinib.

### Immune infiltration analysis and GEO dataset validation

To better elucidate the impact of MECOM, RCN2, and MBNL3 on the tumor microenvironment and immunotherapy, we conducted an immune infiltration analysis. The results show that the expression of MECOM in tumor patients is positively correlated with the infiltration level of CD8 + T cells, neutrophils, and T cell gamma delta. RCN2 was positively correlated with the infiltration level of Macrophage M0 cells, resting Mast cells, B cells, Myeloid dendritic T cells, T cell gamma delta, and CD8 + T cells, and negatively correlated with activated NK cells. The expression of MBNL3 was positively correlated with the infiltration level of CD8 + T cells and neutrophils and B cells (Fig. S5). The above mRNAs were verified in GSE132305 in the GEO database (Fig. S6) and it was found that the expression of MECOM (p-value < 0.01) and RCN2 (p-value = 0.049) was significant, while the expression of MBNL3 was not significant (p-value = 0.12).

## Discussion

In this study, a large number of differentially expressed genes were identified and a ceRNA network was constructed to illustrate potential prognostic and therapeutic targets for CCA. The differentially expressed genes of CCA were enriched with many amino acid kinase-related genes, such as Tyrosine Kinase Inhibitor (TKI), which has been widely used as a targeted therapy for cancers^4^. In addition, in three survival-associated lncRNA-related ceRNA networks, THAP7-AS1 and LINC00519 share the same mRNAs targets, ERBB3 and YWHAZ, both of which are members of the tyrosine kinase family. This indicates that amino acid kinase inhibitors may be a potential strategy to improve the prognosis of CCA.

In the LINC00519/hsa-mir-22-3p/MECOM axis, up-regulated LINC00519 inhibited the expression of hsa-mir-22, thereby promoting the expression of MECOM, which may inhibit the pathway TWEAK/Fn14/NF-kB and promote cell proliferation in CCA^30–32^. Moreover, this network can also inhibit the expression of PTEN protein and activate the PI3K/AKT/mTOR pathway to promote cell metastasis^33^. Moreover, with regard to THAP7-AS1/hsa-mir-155-5p/MBNL3, down-regulated THAP7-AS1 promoted the expression of hsa-mir-155-5p, thereby inhibiting the expression of MBNL3. MBNL3 is involved in cellular selective splicing^34^. Lastly, in the THAP7-AS1/hsa-mir-155-5p/RCN2 relationship, down-regulated THAP7-AS1 promoted the expression of hsa-mir-155-5p, thereby promoting the expression of RCN2. RCN2 might activate the EGFR/ERK pathway by interacting with EGF to cause cell proliferation^35^. In addition, the PI3K/AKT and EGF/EGFR axis have been reported to promote the progression of CCA^36,37^. Combined with current reports on the mechanism of these pathways in CCA and the mechanism of the three genes participating in these pathways in other specific cancers, the roles of three genes in the development of CCA are worthwhile to verify.

It has been reported that LINC00519 could be a potential prognostic biomarker and promote the development of lung adenocarcinoma through positively regulated YAP1, which is a pivotal factor in the Hippo pathway^38,39^ In a recently published report, it was pointed out that THAP7-AS1 can be activated by SP1 transcription, by improving the entry of CUL4B protein into the nucleus to inhibit the expression of miR-22-3p and miR-320a, and it then activated the PI3K/AKT signaling pathway to promote GC progression^40^. Therefore, the mechanism of these three lncRNAs in CCA remains to be experimentally verified. However, based on published reports, LINC00519 and THAP7-AS1 may become potential drug targets and biomarkers for CCA, and the three key networks in which they are involved may be able to explain the occurrence and progress of CCA.

Immune infiltration analysis was performed on the three survival-related genes MECOM, RCN2, and MBNL3, and concerning immune infiltration, the expression level of these three genes in CCA may be related to the degree of infiltration of a variety of immune cells including NK cells and macrophages. Studies have shown that high macrophage infiltration is associated with a poor prognosis, and the activity of NK cells inhibited tumor growth. The increase in NK cells may be a promising method for the treatment of CCA. The high expression of RCN2 in CCA was positively correlated with the degree of infiltration of M0 macrophages and negatively correlated with the appearance of NK cells. Therefore, inhibiting the expression of RCN2 may inhibit the degree of infiltration of M0 macrophages and promote the infiltration of NK cells to improve prognosis. Increased neutrophil infiltration may enhance tumor invasiveness^41^. The expression of MBNL3 was positively correlated with the degree of neutrophil infiltration, suggesting that MBNL3 may affect tumor occurrence by reducing neutrophil infiltration. Changes in the expression of these genes may affect the infiltration of immune cells around tumor tissues, which in turn affects their invasiveness, differentiation, and proliferation.

We also verified three survival-related mRNAs in GSE132305 based on the GEO database and found that MBNL3 down-regulated genes with a p-value > 0.05. This may be due to the complexity of big data sources and the inconsistency of statistical methods. We also supplemented the differential expression analysis based on 8 pairs of paired samples, in which the result of hsa-mir-155 was not significant, which may be due to the small sample size. With only 8 paired samples, some information can be missed. In the case of comparing all CCA samples with all normal samples, the obtained data may be more comprehensive. Therefore, we did not proceed with the analysis based on the results obtained from the paired samples.

We evaluated recent research on these six prognostic genes in specific cancers. MECOM has been reported to be up-regulated and is related to the aggressive behavior of CCA. It may become a potential therapeutic target and classification basis for CCA^42^. It has also been reported that the up-regulation of RCN2 can activate the MYC signal and regulate the activation of the EGFR-ERK pathway to promote the occurrence of hepatocellular carcinoma (HCC)^43^. Furthermore, highly expressed STMN1 has been reported to promote the proliferation of CCA and lead to a poor prognosis^44,45^. In addition, knockdown of MBNL3 can almost eliminate the occurrence of HCC and can up-regulate PXN and promote the occurrence of tumors^21^. Many reports have noted that KIF23 may become a prognostic predictor and biomarker of HCC^46,47^. There is no report on the specific mechanism of MBNL3 and KIF23 in CCA, and only one related study indicated that LONRF3 was a causative gene of pancreatic cancer^48^. As HCC and CCA are close in anatomical position and similar in high-risk factors for carcinogenesis, their pathogenesis may be related. The specific mechanisms of the survival-related genes in the occurrence and development of CCA are not yet clear and have great exploratory value, and may become predictors and biomarkers for the prognosis of patients with CCA.

AZ628, crizotinib, and paclitaxel were predicted to be effective drugs for MECOM, RCN2, and MBNL3, respectively. Among them, AZ628 is a new type of pan-Raf inhibitor, Crizotinib is an ALK tyrosine kinase inhibitor, and paclitaxel acts on the microtubule/tubulin system^49,50^. Whether these treatments can improve CCA patient survival needs further evidence. We evaluated the herbal database for small molecules that may have potential interactions. The small molecules 17-alpha-estradiol, emodin, alpha-tocopherol, and 17-beta-estradiol were identified for molecular docking with MECOM, RCN2, and MBNL3 and all have been reported to inhibit tumor growth. 17-Alpha-estradiol has been shown to inhibit prostate-specific antigen gene expression, which in turn inhibited prostate tumor proliferation and growth^51^. Emodin is a pleiotropic molecule capable of interacting with several major molecular targets, including NF-κB, HER2/neu, HIF-1α, AKT/mTOR, STAT3, CXCR4, p53, and p21. It has been verified to have the ability to treat a variety of cancers^52^. Several reports and clinical trials have confirmed that alpha-tocopherol plays an important role in the treatment and prevention of cancer^53^. 17-Beta-estradiol can inhibit cell growth and induce apoptosis in hepatoma HepG 2 and LCL-PI 11 cell lines^54^ and plays an important role in breast cancer, renal cell carcinoma, prostate cancer, and other cancers. Through molecular docking, we carried out computer-aided verification of the mRNAs downstream of the three key networks we identified, and verified their binding ability with existing anticancer molecules, proving that they had the potential ability to become anticancer drug therapeutic targets.

Compared with our study, Dongkai Zhou et al. predicted the intersection of the lncRNA-miRNA interaction by at least 3 of these databases (miRcode, miRDB, miRanda, miRTarBase, and TargetScan database)^9^. Zhichen Kang et al. downloaded their raw data from the SRA database, which is a possible reason for the discrepancy with our results^10^. The difference in methods between the report of Junyu Long et al. and ours is mainly in the sample size and |logFC| value, which is the main reason for the difference in the results^11^. In the study of Wang Xiang et al., different methods including selected sample size, |logFC| value, and databases for screening lncRNA-mRNA pairs caused differences in results^12^. In addition, the update of the TCGA database was also one of the main reasons for the discrepancies in the obtained lncRNAs related to prognosis. Based on the experience of these studies, we constructed the CCA ceRNA network based on the updated TCGA database, predicted multiple prognostic markers and potential drug targets including MECOM, RCN2, and MBNL3, and carried out molecular docking-assisted verification to provide the basis for the prediction of multiple prognostic markers and potential drug targets. Our research will provide new ideas for CCA-targeted drugs.

There are some limitations to our study. It is undeniable that the limited sample size is a hindrance in the current ceRNA study. We did not verify our results by conducting experiments in vitro and in vivo, but only assisted verification by computer molecular docking. The results of the computer-aided analysis can be validated by in *vitro* cell experiments, and the role of our key ceRNA networks in CCA can be further validated by knocking out key genes (such as MECOM, RCN2, etc.) in CCA cells. At present, many studies have reported on the role of lncRNAs and mRNAs in the above-mentioned key networks in inhibiting liver cancer and CCA. We used molecular docking to verify this possibility and in combination with small molecule anticancer substances, indicating that they may be potential drug targets for CCA. This work can provide ideas for future clinical research and laboratory research.

## Supplementary Information

Supplementary Information 1.

Supplementary Information 2.

Supplementary Information 3.

Supplementary Information 4.

Supplementary Information 5.

Supplementary Information 6.

Supplementary Information 7.

Supplementary Information 8.

Supplementary Information 9.

Supplementary Information 10.

## Data Availability

The datasets generated during the current study are available from the corresponding author upon reasonable request.

## Abbreviations

CCA: Cholangiocarcinoma
EBRT: External beam radiation therapy
lncRNAs: Long non-coding RNAs
ceRNA: Competitive endogenous RNA
miRNAs: MicroRNAs
mRNAs: Messenger RNAs
TCGA: The cancer genome atlas
PCA: Principal component analysis
GO: Gene ontology
GDSC: The genomics of drug sensitivity in cancer
95%CI: 95% Confidence interval
HR: Hazard ratio
LINC00519: Long intergenic non-protein coding RNA 519
THAP7-AS1: THAP7 antisense RNA 1
KIF23: Kinesin family member 23
STMN1: Stathmin 1
MECOM: MDS1 and EVI1 complex locus
RCN2: Reticulocalbin-2
LONRF3: LON peptidase N-terminal domain and ring finger 3
MBNL3: Muscle blind like splicing regulator 3
AUC: Area under the curve
TKI: Tyrosine kinase inhibitor
HCC: Hepatocellular carcinoma
