# Supplementary figures and images for: Identification of a prognosis-related ceRNA network in cholangiocarcinoma and potentially therapeutic molecules using a bioinformatic approach and molecular docking
Source: Sci Rep. 2022 Sep 28;12:16247. doi: 10.1038/s41598-022-20362-w (PMC9519560; doi:10.1038/s41598-022-20362-w)

A

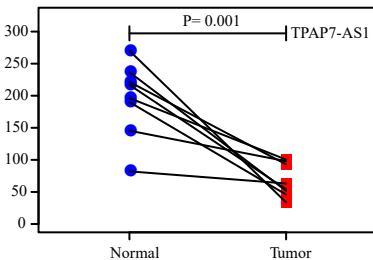

B

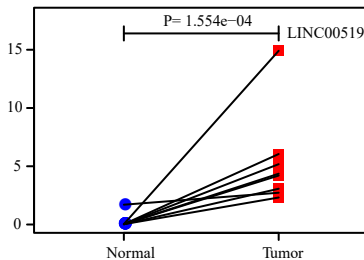

C

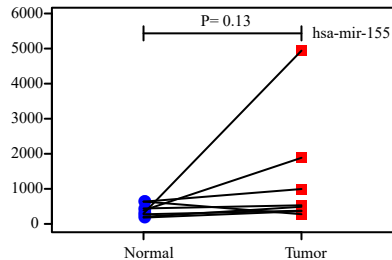

D

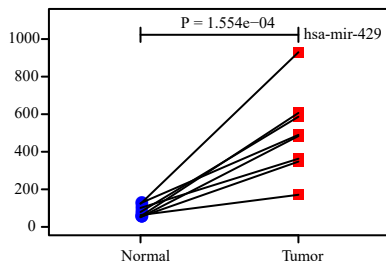

E

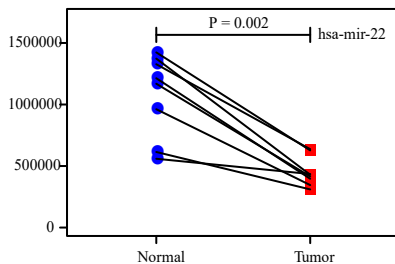

F

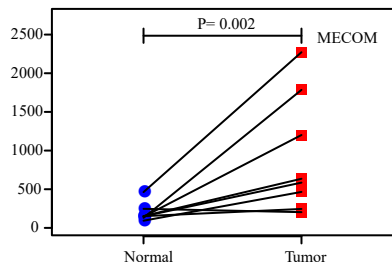

G

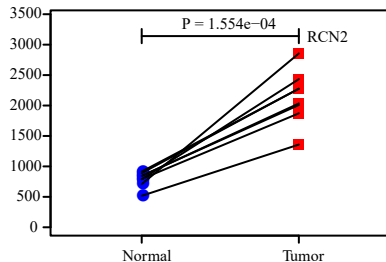

H

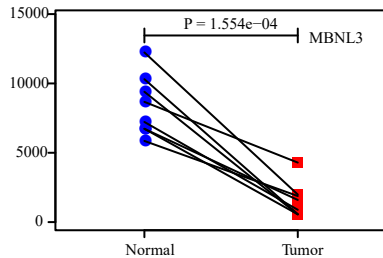

Supplement: Supplementary file 1 — Supplementary Information 1. [file 41598_2022_20362_MOESM1_ESM.pdf]

A

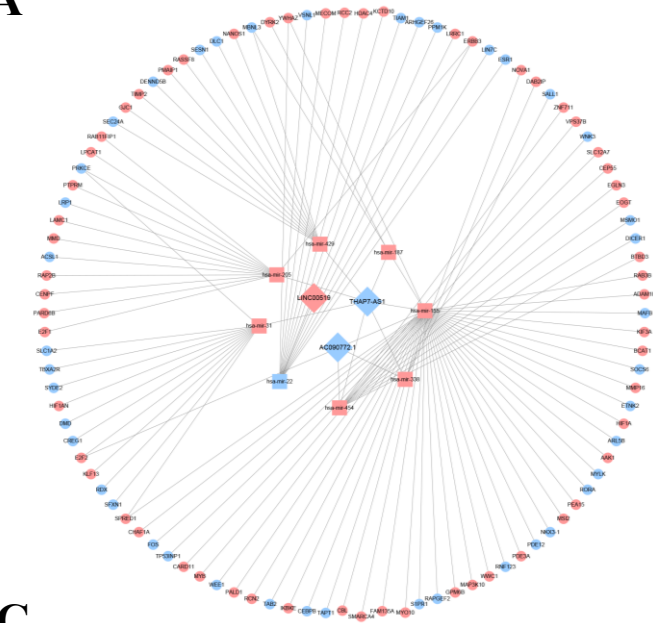

C

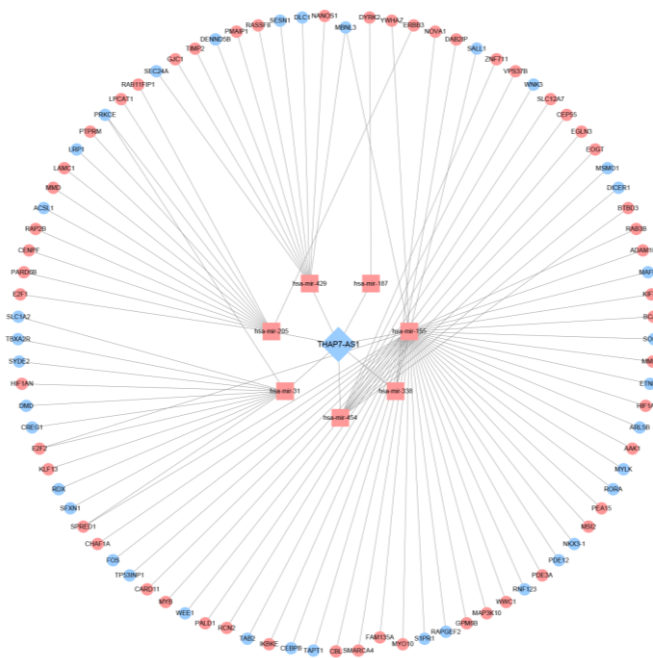

B

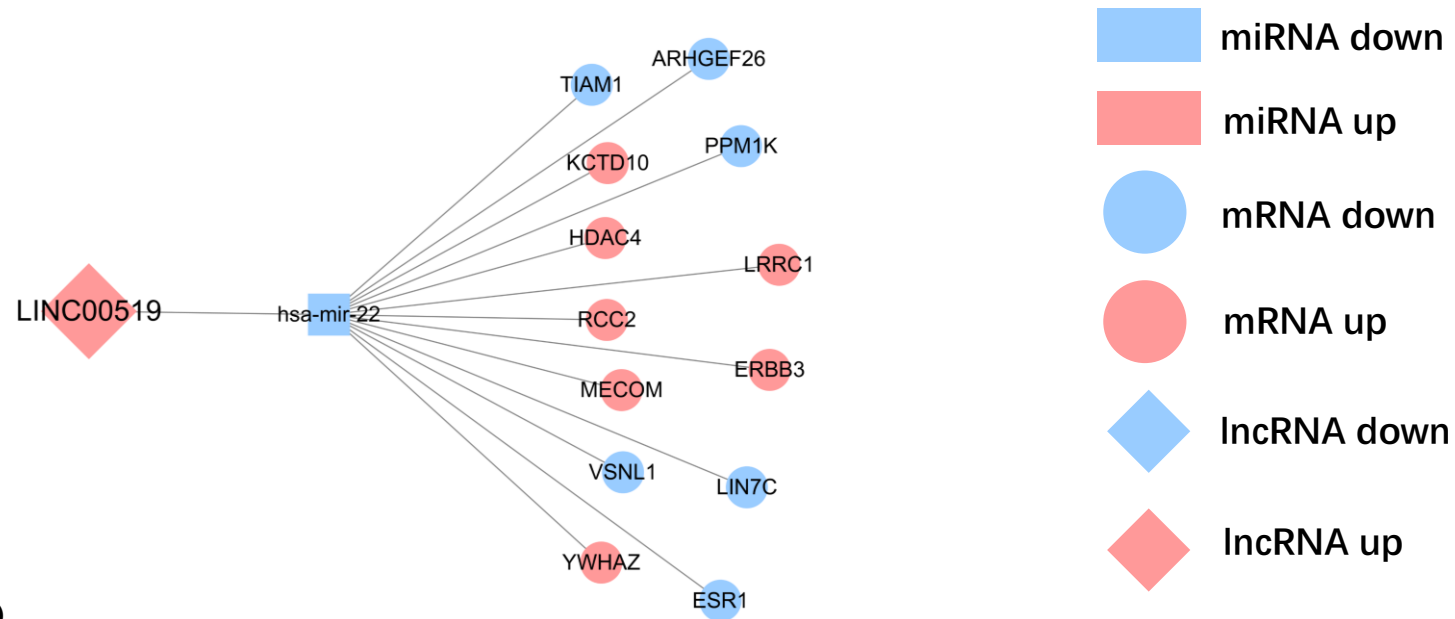

D

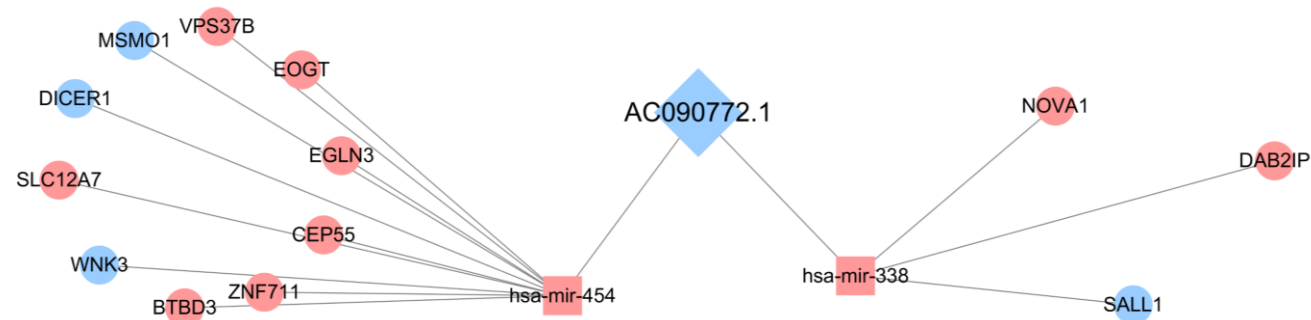

Supplement: Supplementary file 2 — Supplementary Information 2. [file 41598_2022_20362_MOESM2_ESM.pdf]

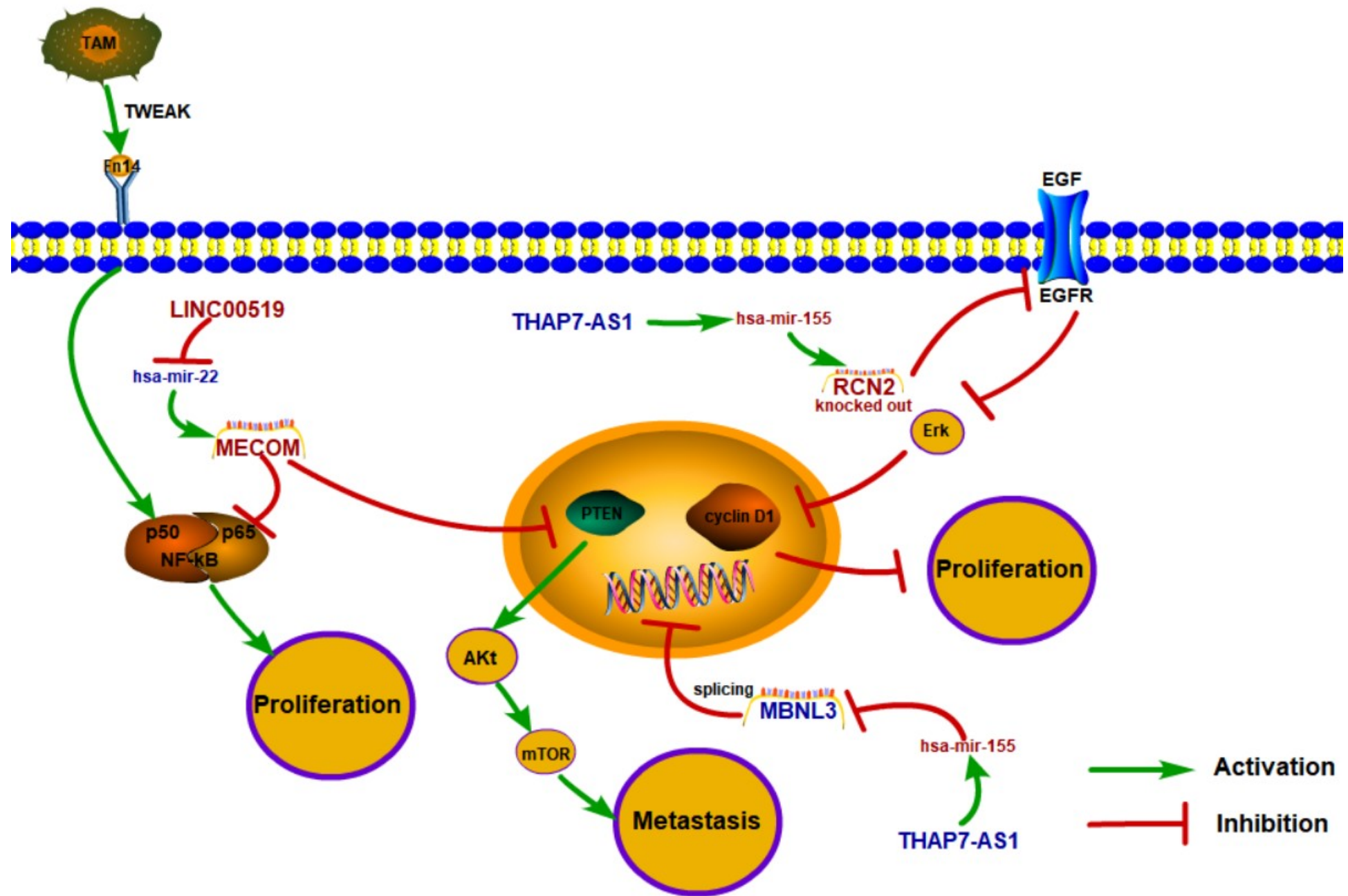

Supplement: Supplementary file 3 — Supplementary Information 3. [file 41598_2022_20362_MOESM3_ESM.pdf]

**A**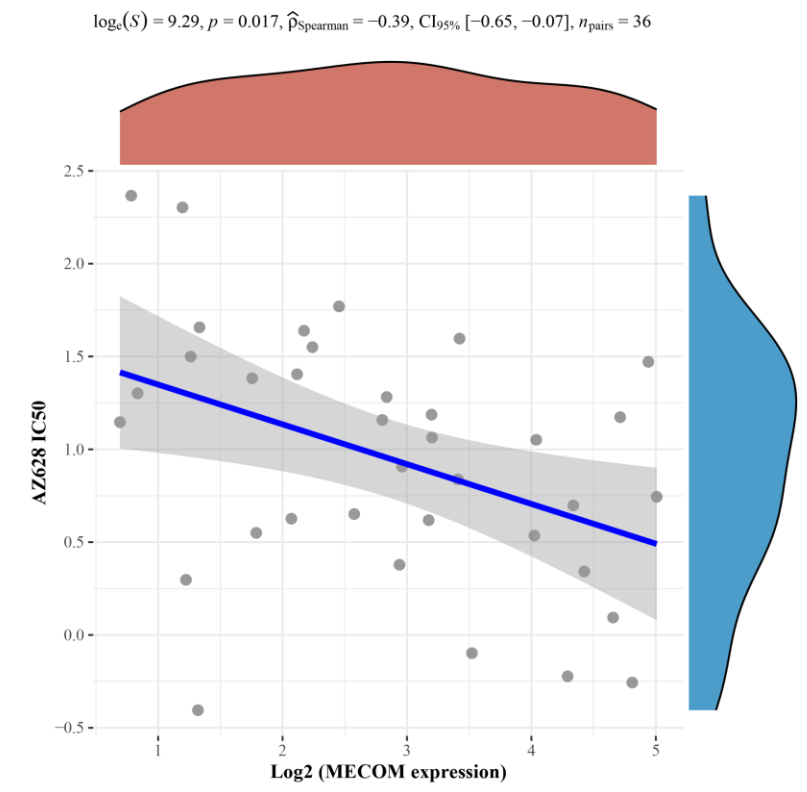**B**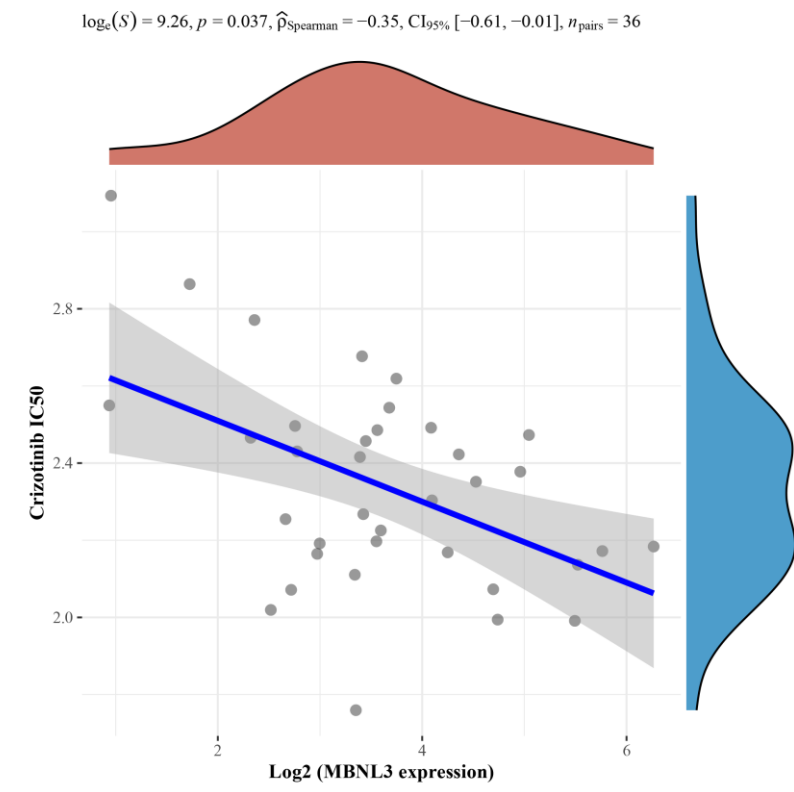**C**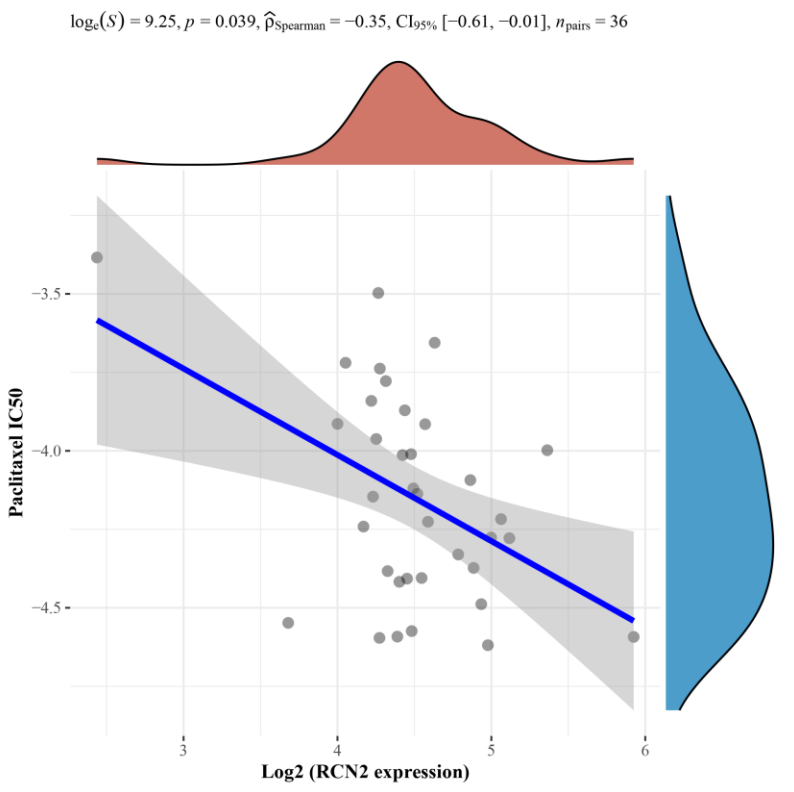

Supplement: Supplementary file 4 — Supplementary Information 4. [file 41598_2022_20362_MOESM4_ESM.pdf]

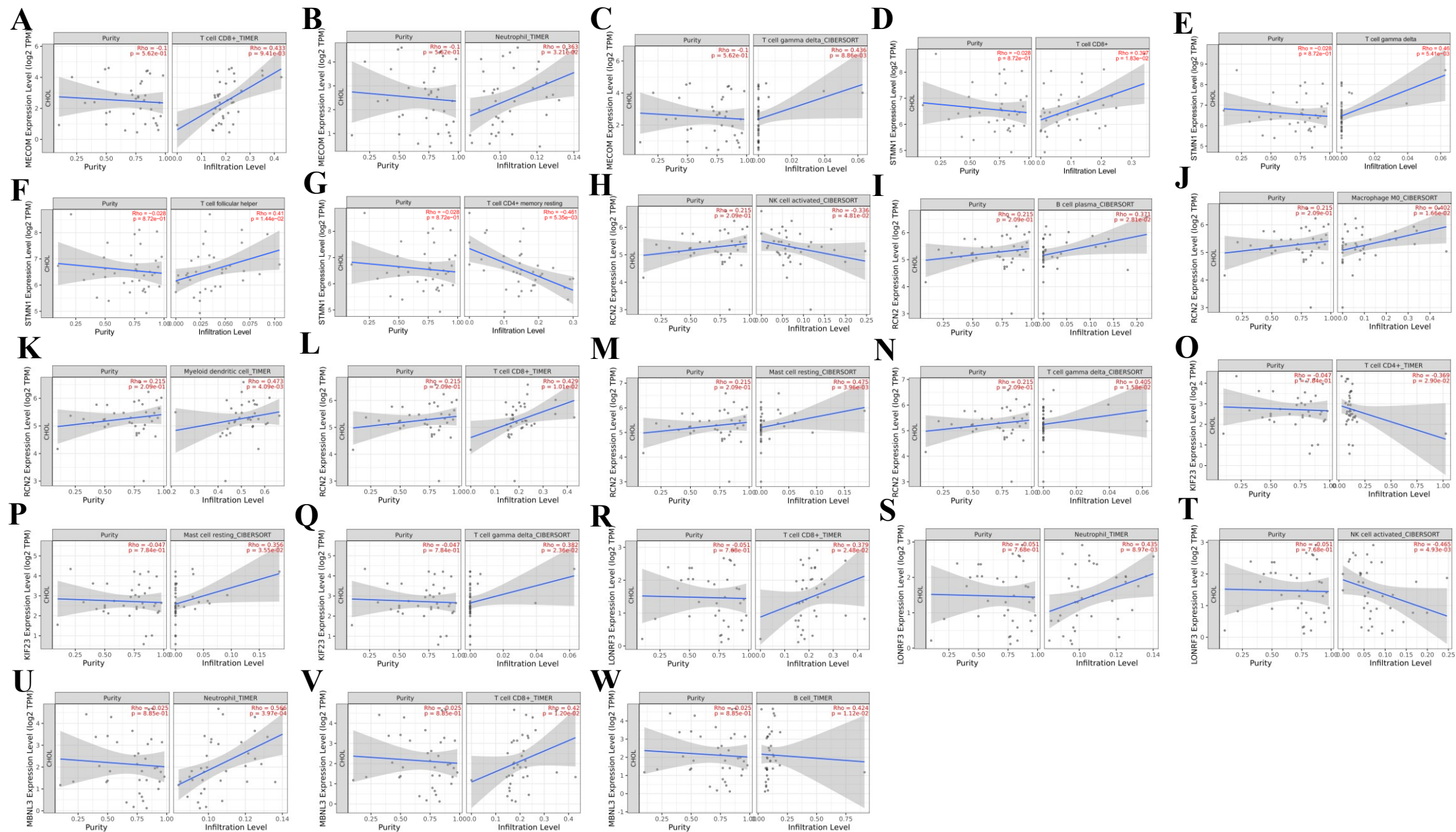

Supplement: Supplementary file 5 — Supplementary Information 5. [file 41598_2022_20362_MOESM5_ESM.pdf]

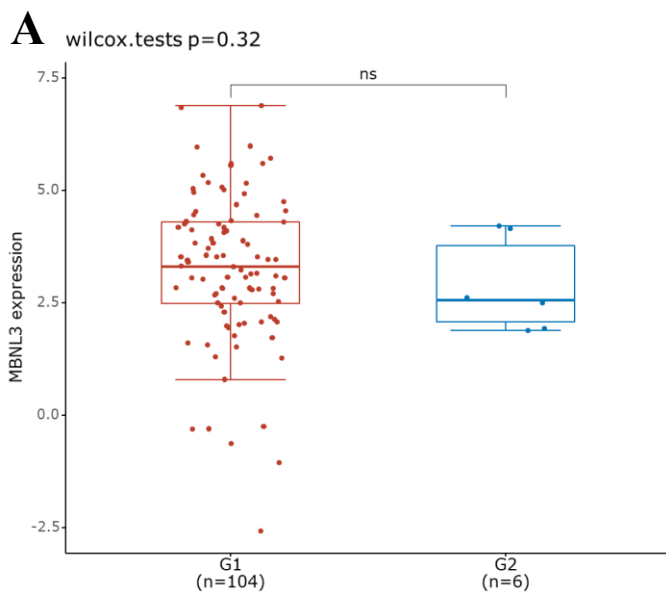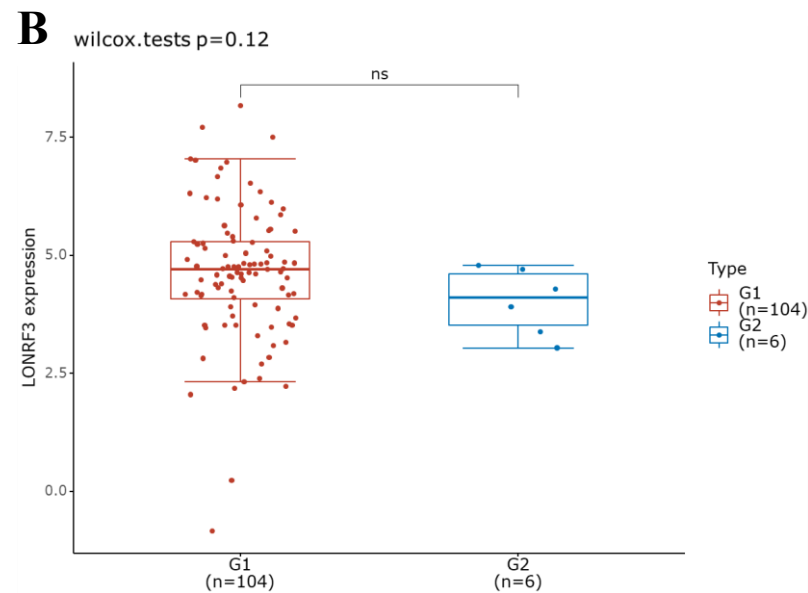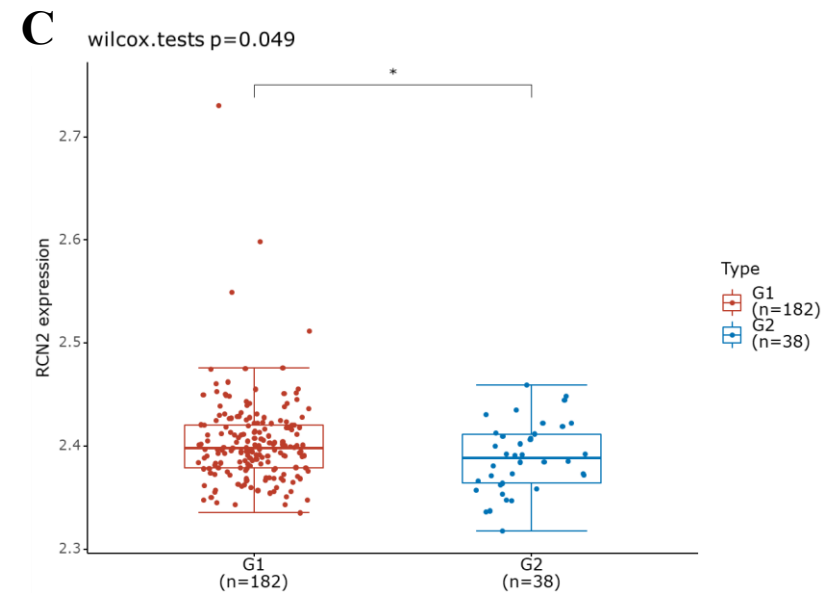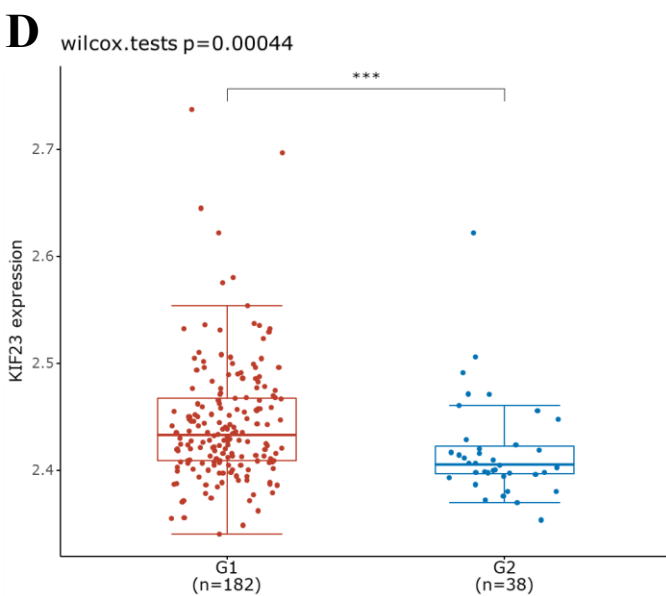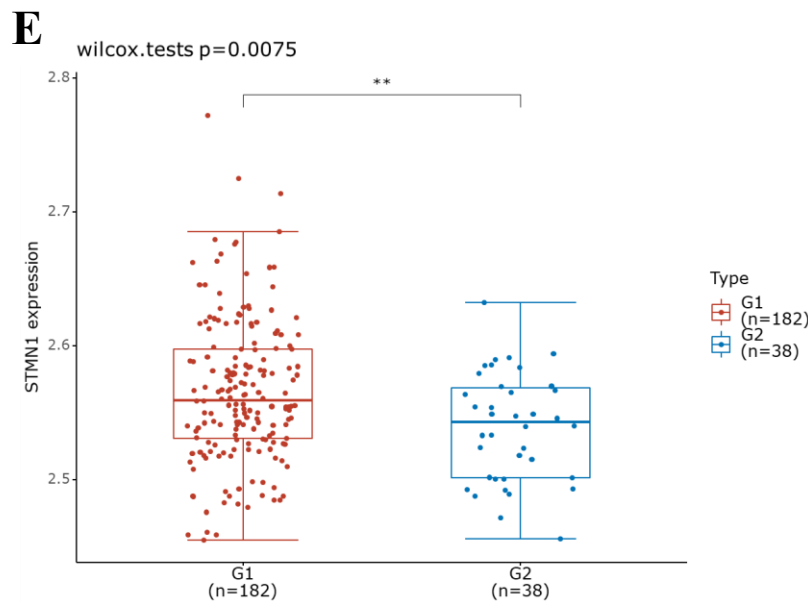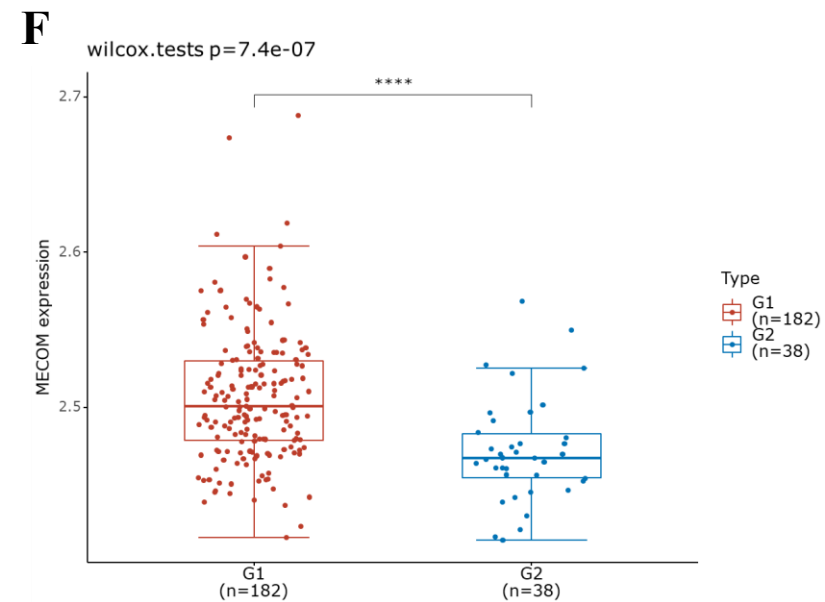

Supplement: Supplementary file 6 — Supplementary Information 6. [file 41598_2022_20362_MOESM6_ESM.pdf]
