# Supplementary material for: Identification of a prognosis-related ceRNA network in cholangiocarcinoma and potentially therapeutic molecules using a bioinformatic approach and molecular docking
Source: Sci Rep. 2022 Sep 28;12:16247. doi: 10.1038/s41598-022-20362-w (PMC9519560; doi:10.1038/s41598-022-20362-w)
Supplement: Supplementary file 7 — Supplementary Information 7. [file 41598_2022_20362_MOESM7_ESM.docx]

**FigureS1** The differential analyses of key ceRNA networks based on 8 paired samples of CCA available from the TCGA database. (A): THAP7-AS1; (B): LINC00519; (C): hsa-mir-155; (D): hsa-mir-429; (E): hsa-mir-122; (F): MECOM; (G): RCN2; (H): MBNL3.

**FigureS2** The ceRNA networks based on lncRNAs with significant survival; (A): The construction of ceRNA network with three lncRNAs and their downstream DEmiRNAs and DEmRNAs; (B): ceRNA network constructed around LINC00519; (C): ceRNA network constructed around THAP7-AS1, (D) ceRNA network constructed around AC090772.1; the shape represented the type of RNA, blue meant down-regulation in cancer samples, and red meant up-regulation in cancer samples.

**FigureS3** The possible pathways that downstream genes of three ceRNA networks participated in the occurrence and development of cancer based on the summary of existing reports; the green arrow represented promotion, the red line represented block. Drawn by pbtools version 2.0, <URL: https://pbtools.software.informer.com/2.0/>

**FigureS4** Drug sensitivity analysis of specific genes and the significant IC50 curves (p-value<0.05). (A): MECOM and AZ628, (B): MBNL3 and crizotinib (C): RCN2 and paclitaxel

**FigureS5** The relationship between the expression of 6 genes in CCA samples and the degree of specific immune cell infiltration (P value< 0.05). MECOM(A-C), STMN1(D-G), RCN2(H-N), KIF23(O-Q), LONRF3(R-T), MBNL3(U-W)

**FigureS6** The differential expression verification of MBNL3, LONRF3, RCN2, KIF23, STMN1, MECOM (A-F) between cancer samples and control samples based on the GEO database; different colors represented different sample groups.
