# Supplementary material for: Identification of a prognosis-related ceRNA network in cholangiocarcinoma and potentially therapeutic molecules using a bioinformatic approach and molecular docking
Source: Sci Rep. 2022 Sep 28;12:16247. doi: 10.1038/s41598-022-20362-w (PMC9519560; doi:10.1038/s41598-022-20362-w)
Supplement: Supplementary file 8 — Supplementary Information 8. [file 41598_2022_20362_MOESM8_ESM.docx]

**Table S1 The prediction of lncRNA-miRNA interactions**

The prediction of miRNA-lncRNA interactions based on the miRcode database

**Table S2 The prediction of miRNA-mRNA interactions**

The prediction of miRNA-mRNA interactions that meet the conditions of three databases (the miRDB database, Target Scan Human database**,** and miRtarBase database) simultaneously
